# Elevated levels of matrix metalloprotein-3 in patients with coronary aneurysm: A case control study

**DOI:** 10.1186/1468-6708-5-10

**Published:** 2004-10-13

**Authors:** Istemihan Tengiz, Ertugrul Ercan, Emil Aliyev, Cevad Sekuri, Can Duman, Imre Altuglu

**Affiliations:** 1 Central Hospital, Cardiology Department, Izmir, Turkey; 2 Kent Hospital, Cardiology Department, Izmir, Turkey; 3 Kocaeli University Medical School, Biochemistry Department, Kocaeli, Turkey; 4 Ege University Medical School, Microbiology Department, Izmir, Turkey

## Abstract

**Background:**

Matrix metalloproteinases (MMPs) have been implicated in the pathogenesis of arterial aneurysms through increased proteolysis of extracellular matrix proteins. Increased proteolysis due to elevated matrix degrading enzyme activity in the arterial wall may act as a susceptibility factor for the development of coronary aneurysms. The aim of this study was to investigate the association between MMPs and presence of coronary aneurysms.

**Methods:**

Thirty patients with aneurysmal coronary artery disease and stable angina were enrolled into study (Group 1). Fourteen coronary artery disease patients with stable angina were selected as control group (Group 2). MMP-1, MMP-3 and C-reactive protein (CRP) were measured in peripheral venous blood and matched between the groups.

**Results:**

Serum MMP-3 level was higher in patients with aneurismal coronary artery disease compared to the control group (20.23 ± 14.68 vs 11.45 ± 6.55 ng/ml, p = 0.039). Serum MMP-1 (13.63 ± 7.73 vs 12.15 ± 6.27 ng/ml, p = 0.52) and CRP levels (4.78 ± 1.47 vs 4.05 ± 1.53 mg/l, p = 0.13) were not significantly different between the groups.

**Conclusion:**

MMPs can cause arterial wall destruction. MMP-3 may play role in the pathogenesis of coronary aneurysm development through increased proteolysis of extracellular matrix proteins.

## Introduction

Coronary artery aneurysms are defined as dilated coronary artery segments that are greater than 1.5 times the diameter of adjacent normal segments [1,2]. The gold standard for diagnosing this type of aneurysm is coronary angiography, which provides information about the size, shape, location and number of aneurysms.

Coronary aneurysms may occur during the development of coronary atherosclerosis. Previous studies have shown that coronary aneurysms are observed in 1% to 5% of patients with angiographic evidence of coronary artery disease [3-6]. In some studies, coronary aneurysms have been associated with an increased risk of myocardial infarction [3,4]. Although the mechanisms responsible for coronary aneurysm formation during the atherosclerotic process are unclear, atherosclerosis-induced aneurysms derive primarily from thinning and/or destruction of the media [6-8].

Possible factors contributing to aneurysms are matrix-degrading enzymes such as collagenases, gelatinases, and stromelysins [9,10]. More specifically, matrix metalloproteinases (MMPs) are enzymes that can degrade the structural proteins of connective tissue. Degradation of extracellular matrix proteins may weaken the connective tissue, thereby leading to a weakened vascular wall.

We investigated the association between MMPs and coronary artery aneurysm by measuring the levels of MMP-1 and MMP-3 (both of which represent markers of proteolytic activity) in patients with coronary artery disease, some of whom had coronary aneurysms (cases) and others who did not (controls).

## Methods

### Patient population

We reviewed the medical records of patients who had undergone coronary angiography between January, 2002 and April, 2003. Among 4,456 cases reviewed, 55 patients (1.23%) diagnosed with aneurysmal coronary artery disease were selected. Sixteen patients with acute coronary syndromes and nine patients with balloon angioplasty history were excluded from the study. The remaining 30 patients with aneurysmal coronary artery disease patients were enrolled into the study. Transverse diameter of an aneurysm and reference vessel were measured using the post-processing software (Schimadzu Corporation, DIGITEX ALPHA Plus System, Kyoto, Japan, 2001). The ratio between dilated coronary artery segment and reference vessel diameter was calculated. The control patients (n = 14) had coronary artery disease, but were free of aneurysmal coronary dilatation. Both groups had positive exercise stress tests and had been diagnosed with stable angina. Blood biochemistry and echocardiography were performed in all patients. No patient had a history of coronary atherectomy or balloon angioplasty. All participants gave informed consent.

Autoimmune disease, inflammatory arteritis, chronic or, acute infectious disease, use of steroid or anti-inflammatory drugs within the last three months, renal failure and cancer were accepted as exclusion criteria.

### Laboratory assays

#### Specimen collection

Fasting blood samples (8–10 hours fast) were obtained from the antecubital vein at approximately 9:00 a.m. These were centrifuged for 10 min at 3,000 × g at a temperature of about 4°C. Serum was stored at -70°C. Blood samples were analyzed at the Ege University Department of Microbiology, Section of Serology.

#### Assay protocol for MMP-1 and MMP-3

MMP levels were determined using enzyme-linked immunosorbent assay (ELISA) kits, according to the manufacturer's instructions (MMP-1, Biotrak Amersham Pharmacia Biotech, United Kingdom; RPN 2610; MMP-3, Biotrak Amersham Pharmacia Biotech, United Kingdom; RPN 2613). The ELISA kit measured total MMP-1 (pro MMP-1, free MMP-1, MMP1/tissue inhibitor MP-1 complex), total MMP-3 (pro MMP-3, free MMP-3, MMP3/tissue inhibitor MP-1 and MMP3/tissue inhibitor MP-2 complex) at >89% cross reactivity. Samples were incubated in microtitre wells pre-coated with anti-MMP-1 (lyophilized rabbit anti-MMP-1) and anti-MMP-3 (peroxidase labelled Fab antibody to MMP-3) antibodies. The assays use the pro form of a detection enzyme that can be activated (by captured active MMP) into an active detection enzyme. MMP-1 and MMP-3 can be measured in the range of 6.25–100 ng/ml and 3.75–120 ng/ml, respectively. The results received from the optic scanners at 450 nm were converted into ng/ml values from a standard curve. All samples were run in duplicate and were averaged. Within-assay precision values for duplicate determinations were 5.5%, 7.9% and 7.3% at MMP-1 concentrations of 16.89 ± 0.94 ng/ml, 35.53 ± 2.82 ng/ml and 54.08 ± 4.0 ng/ml, respectively. Between-assay precisions for repeated measurements of the same sample were 11.6%, 12.0% and 13.2% at MMP-1 concentrations of 23.19 ± 2.68 ng/ml, 55.27 ± 6.65 and 98.04 ± 12.93, respectively. The within-assay precisions for duplicate determinations were 4.8%, 2.4% and 2.1% at MMP-3 concentrations of 13.7 ± 0.66 ng/ml, 33.7 ± 0.83 ng/ml and 83.2 ± 1.76 ng/ml, respectively. Between-assay precisions for repeated measurement of the same sample were 13.3%, 11.7% and 8.8% at MMP-3 concentrations of 11.2 ± 1.49 ng/ml, 27.6 ± 3.24 and 75.4 ± 6.63, respectively.

#### Determination of C-reactive protein levels

Serums were obtained by centrifugation of vacutainer-clotted tubes at 3,000 rpm for 10 minutes. High sensitivity C-reactive protein (hs-CRP) samples were stored at -30°C and analyzed by latex particle-enhanced immunoturbidimetric assay. The total median inter-assay and intra-assay coefficients of variation for the assays were <6% for CRP. All results were recorded in the patients' files.

### Statistical analyses

All values are reported as mean ± SD. Chi Square test was used in the comparison of categorical variables while student unpaired-t test or Mann-Whitney Rank Sum tests were used, where appropriate, in the univariate analysis. Statistical analyses were performed with SPSS statistical software. A value of p < 0.05 was considered to be statistically significant.

## Results

There were no significant differences in baseline characteristics between cases and controls. High-density lipoprotein, low-density lipoprotein, total cholesterol and triglyceride levels were not statistically different between the groups. Clinical characteristics of and medication use by the groups are shown in Table 1.

**Table 1:** Clinical Characteristics and Medication Use of Study Participants

	**Group 1 (n = 30)**	**Group 2 (n = 14)**	**p**
**Mean age (yrs)**	55.2 ± 10.0	51.8 ± 7.7	NS
**Male sex % (n)**	70%(21)	64%(9)	NS
**Diabetes Mellitus % (n)**	13%(4)	14%(2)	NS
**Hypertension % (n)**	30%(9)	21%(3)	NS
**Smoking % (n)**	60%(18)	50%(7)	NS
**TC (mg/dl)**	196.8 ± 31.7	195.1 ± 38.2	NS
**TG (mg/dl)**	148.7 ± 71.2	151.7 ± 64.0	NS
**HDL-C (mg/dl)**	46.7 ± 11.8	50.7 ± 13.0	NS
**LDL-C (mg/dl)**	125.1 ± 28.2	116.7 ± 34.2	NS
**hs-CRP (mg/L)**	4.78 ± 1.47	4.05 ± 1.53	NS
**MMP-1 (ng/ml)**	13.63 ± 7.73	12.15 ± 6.27	NS
**MMP-3 (ng/ml)**	20.23 ± 14.68	11.45 ± 6.55	0.039
**Baseline therapy**			
Aspirin	73%(22)	64%(9)	NS
Nitrate	57%(17)	64%(9)	NS
Statin	17%(5)	21%(3)	NS
**Number of stenotic vessels**			
One vessel disease	37%(11)	36%(5)	NS
Two vessel disease	50%(15)	43%(6)	NS
Three vessel disease	13%(4)	21%(3)	NS
**Reference vessel diameter (mm)**	2.95 ± 0.48	-	-
**Aneurysm vessel diameter (mm)**	4.78 ± 0.93	-	-
**Aneurysm/reference vessel ratio**	1.6 ± 0.1	-	-
**Aneurysm segment**			
Right coronary artery	53%(16)	-	-
Left anterior descending artery	27%(8)	-	-
Left Circumflex artery	30%(9)	-	-

**Group 1:**Patients with coronary aneurysm, **Group 2:**Patients without coronary aneurysm, **TC:**Total cholesterol, **TG:**Triglyceride, **HDL-C:**High-density lipoprotein cholesterol, **LDL-C:**Low-density lipoprotein cholesterol, **hs-CRP:**High sensitivity C- reactive protein, **MMP-1:**Matrix metalloproteinase-1, **MMP-3:**Matrix metalloproteinase-3, **NS:**Non-significant

**Figure 1:**
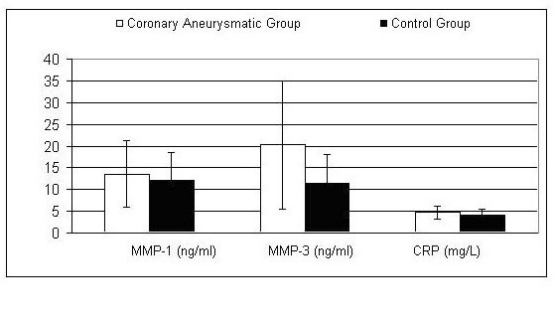
text

Mean serum MMP-1 (13.63 ± 7.73 vs 12.15 ± 6.27 ng/ml, p = 0.52) and CRP levels (4.78 ± 1.47 vs 4.05 ± 1.53 mg/l, p = 0.13) were not significantly different between cases and controls. Mean serum MMP-3 values were significantly higher in the cases than in controls (20.23 ± 14.68 and 11.45 ± 6.55 ng/ml respectively, p = 0.039). MMP-1, MMP-3 and hs-CRP levels are shown in Figure 1.

## Discussion

Essential factors contributing to the formation of coronary aneurysms include vessel media degradation and ulceration due to increased proteolytic activity. Connective tissue integrity, another factor contributing to aneurysm development, depends on the balance between degradation and repair of the extracellular matrix. Activation or inhibition of degrading enzymes affects extracellular matrix modeling [9,10], which, in turn, affects connective tissue and vascular wall integrity.

Matrix-degrading enzyme activity is a tightly controlled process that involves transcription, activation of latent pro-enzymes and inhibition of proteolytic activity [11-13]. A key step in the regulation of MMPs may occur at the level of transcription [14]. The mechanism by which gene transcription is mediated is thought to involve a prostaglandin E_2_(PGE_2_)-cAMP- dependent pathway. G-proteins have been implicated in this pathway [15]. Transcription activity can be stimulated by a variety of inflammatory cytokines, hormones, and growth factors [16-19]. Several factors are also known to inhibit MMP gene expression and these include indomethacin, corticosteroids, and interleukin-4 [17,20,21].

MMP activity is also regulated by tissue-specific inhibitors. There are four known tissue inhibitors of metalloproteinases (TIMP-1, -2, -3 and -4). The TIMPs are secreted by a variety of cell lines, including smooth muscle cells and macrophages. Their activity is increased by growth factors and either increased or decreased by different interleukins [22]. Increased levels of MMP-2, MMP-3, MMP-9 and MMP-12 have been identified in aneurysm vessel walls [23-27]. Gene disruption of MMP-9 suppresses the development of experimental abdominal aortic aneurysms [28]. Conversely, decreased levels of TIMPs have been found in the aneurysm wall [26]. Allaire et al. [29] reported that local expression of TIMP-1 may prevent aortic aneurysm degeneration and rupture in a rat model. Carrell et al. [30] examined differences in MMPs between patients with aortic aneurysm and patients with aortic atherosclerosis but without aneurysm. Among a wide range of MMPs tested, only MMP-3 was over-expressed in the aortic aneurysm samples. Reduced aneurysm formation has been observed in mice with MMP-3 gene inactivation [31]. Finally, the recent observation that high circulating levels of MMP-3 are associated with coronary lesions in Kawasaki disease [32] also supports an important role for MMP-3 in the pathogenesis of coronary aneurysms. These data suggest that proteolytic balance in the vascular wall plays a key role in aneurysm development.

MMP-1 (interstitial collagenase) and MMP-3 (stromelysin-1) are members of a family of proteinases that degrade one or more components of the extracellular matrix. In our study, it appears that elevated MMP-3 activity may represent a risk factor for coronary aneurysm formation. This finding is concordant with previously published studies. The mechanisms underlying this association are unclear. MMP-3 gene disruption may be responsible. Lamblin et al. [33] have reported similar findings, namely, that the MMP-3 5A allele is associated with the occurrence of coronary aneurysm.

Others have reported that MMP-3 is expressed in atherosclerotic plaque cells, but not by cells in normal arteries [34-37]. In addition, extensive inflammation and destruction of musculo-elastic vessel wall elements have been observed in dilated human coronary arteries [38,39]. Schoenhagen et al. [40] suggest that the degradation of extracellular matrix by MMP-3 may contribute to the expansion of the coronary vessel wall. This effect is characteristic of positive remodeling. Based on these and our own observations, we maintain that MMP-3 over-expression may occur in aneurysm segments. Histopathologic studies would be needed to clarify whether or not this is the case.

MMP levels are elevated in patients with acute myocardial infarction, unstable angina and coronary angioplasty [35,41,42]. All patients in our study had been diagnosed with stable angina before being enrolled into the study.

CRP reflects systemic inflammatory activity. In this study, we did not observe increased CRP levels in those patients with coronary aneurysms. One explanation for similar CRP expression between cases and controls might be that all study subjects had been diagnosed with stable angina pectoris.

Varying degrees of inflammation are reported among individuals with abdominal aortic aneurysms. This variation may relate to possible confounding due to clinical manifestations (asymptomatic or symptomatic) and aneurysm progression rates (cm/year). Other investigators have failed to observe increased CRP levels among asymptomatic patients with abdominal aortic aneurysm [43].

Because elevated MMP-3 levels likely contribute to the development of coronary aneurysms, this matrix-degrading enzyme may represent an important therapeutic target. Luan et al. [44] reported that a number of statins inhibit MMP-3 activity in rabbits. COX-2 inhibitors may also suppress MMP expression. Production of MMPs by macrophages occurs through a PGE_2_/cAMP-dependent pathway [45]. Theoretically, COX-2 inhibitors could attenuate this pathway. Another target of MMP inhibition has been demonstrated in animal models of adenovirus-mediated TIMP gene transfer [46].

In reporting our findings, we acknowledge that measurement of TIMP levels between cases and controls would have provided useful information about the possibility of proteolytic imbalance. Similarly, measurement of locally produced inflammatory cytokines, hormones and growth factors would be interesting to know about, since these regulate matrix-degrading enzyme expression [16-19]. This could provide relevant information, as systemic inflammatory activity may not reflect local inflammatory infiltration in aneurysm segments. Finally, the study would have benefited from having a larger sample size as well as genotype determination.

We conclude that MMP-3 overexpression due to a proteolytic imbalance may lead to coronary aneurysm development through degradation of matrix components, especially lamina elastica. New medical therapeutic options targeted specifically against MMP-3 may prove useful in the prevention of aneurysm formation.
